# New products

**DOI:** 10.1155/S1463924690000220

**Published:** 1990


Journal of Automatic Chemistry, Vol. 12, No. 4 (July–August 1990), pp. 174–182

### New products

### Sartec

Sartec are making step changes to the way in which they operate in order to facilitate expansion plans. During the seven years since the company was established, the market for pollution monitoring equipment has grown rapidly and with it Sartec's range of instruments, applications and expertise. This growth continues unabated on all three fronts.

New instrumentation soon to be released will include a high-temperature total organic carbon analyser and volatile organics analyser.

To alleviate pressure on technical staff in future some service work will be placed with approved Sartec agents on a subcontract basis, a move which will benefit customers by providing a faster response to their needs.

The company is also making inroads into Europe with the imminent opening of an office in Oslo which will service the oil, pulp and paper and chemical industries.

To keep customers up to date with progress, the first in a new series of Sartec Newsletters will appear in April.

*Sartec are based at Bourne Industrial Estate, Wrotham Road, Borough Green, Sevenoaks, Kent TN15 8DG, UK. Tel.: 0732 884815.*

### Moisture analyser

The new M30 Moisture Analyser, which will operate in either automatic or manual mode, will provide read-outs of % moisture, % dry weight, % atro 1, % atro 2, analysis time and the temperature setting. All read-outs are displayed on a large LCD display, with facilities available for keeping a permanent record.

The MA30 was designed specifically for a single application, that of fast and easy moisture determination in tough environments, hence Sartorius is able to bring the balance to market at the very competitive price of £1495. Using an infrared dark beam means that drying times are a few minutes, compared with several hours in a drying oven. Another feature of the unit is the non-volatile memory which prevents loss of read-out in the event of a mains failure.

Moisture determination is a broad field and at Sartorius it is defined as the use of measurement technology to determine the moisture content of the dry weight of a liquid or a solid in samples typically weighing just a few grammes. The MA30's high heating capability allows it to produce results in a few minutes, even for samples which do not release moisture very easily. The temperature span is adjustable from 40 °C to 160 °C in increments of 5 °C.

In the automatic mode, the unit shuts itself off as soon as the sample is 'dry', to avoid burning. Alternatively, the unit can be used in a manual mode where pre-set heating

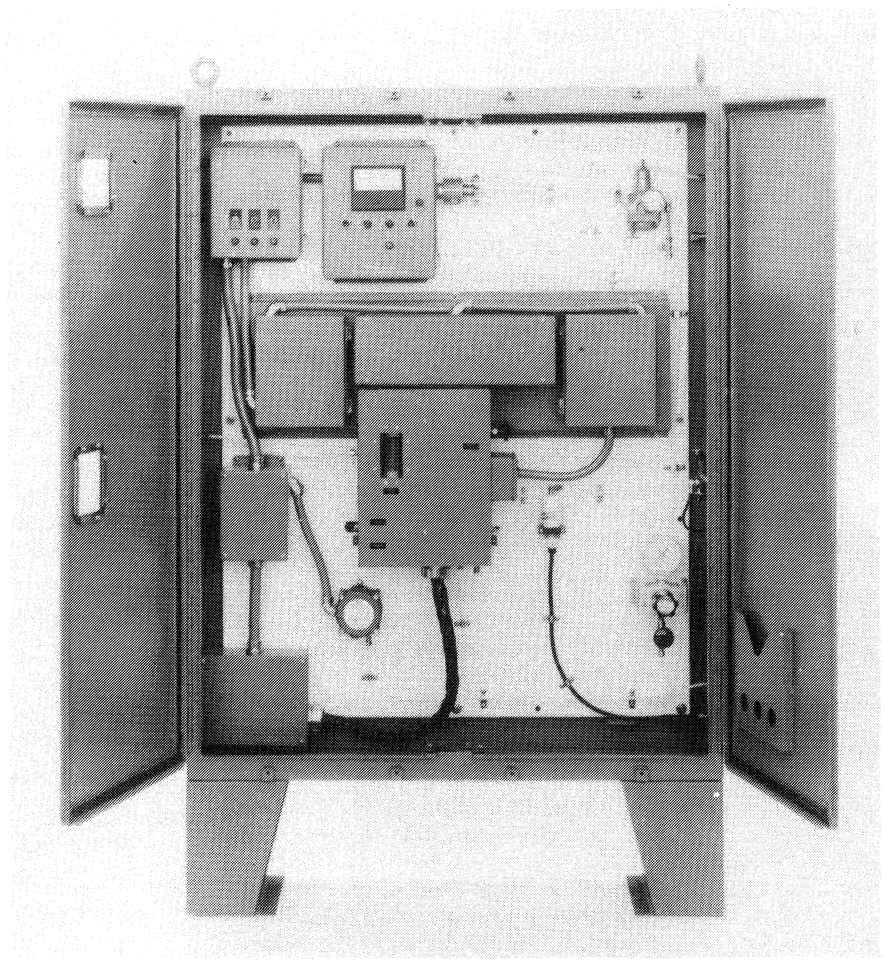

*Teledyne's Model 691 Sulfur Dioxide ($SO_2$) Analyser System provides accurate on-line monitoring of flue gas in boilers, furnaces and virtually any combustion process. The 691 immediately alerts plant operators to combustion conditions that will result in excessive $SO_2$ emissions. The 691's sample conditioning system removes particulates and provides precise temperature control of the sample assuring sample integrity and optimum accuracy. The single chopped-beam, dual-wavelength, electro-optical photometric analyser achieves high sensitivity and fast response. Outstanding long-term stability is achieved through a fully automatic zero. Details from The Harlequin Centre, Southall Lane, Southall, Middlesex UB2 5NH, UK. Tel.: 081 571 9596.*

times can be programmed. The new MA30 can be programmed to switch off after a preset time between 0·1 to 99 min.

The new MA30 Moisture Analyser's standard interface enables a print-out to be obtained of the initial, intermediate and final readings.

*For further information contact: Peter Butler, Sartorius UK Ltd, Weighing Division, Longmead Business Centre, Blenheim Road, Epsom, Surrey KT19 9QN, UK. Tel.: 03727 45811.*

### Automated luminescence counting

The Bio-Orbit Type 1251 Luminometer presents a sophisticated and fully automatic method of conducting rapid luminescence assays in applications as diverse as microbiology and biochemistry; but with the increasing emphasis on cleaning-up the food chain, it is also enabling quality control to be tightened during the manufacturing and distribution process. With a Type 1251 luminometer it is possible to count bacterial numbers, which would take at least 18 h to complete using conventional plate counting procedures, in only 15 min.

The operation is user-friendly and can be controlled by several PCs in common use to give very precise and reproducible incubation time, temperature, reagent addition, degree of mixing and even the reading mode, all of which can be pre-set. Up to 300 samples/h can be processed using a 25 position carousel which has sophisticated temperature control and thus acts as an incubator, as well as a sample handler. Precise micro-volumes can be dispensed automatically from up to three Type 1291 dispensers directly into the sample whilst it is in the thermally controlled measuring chamber. The reagents are pre-heated before their addition to the sample tube by a heat exchanger.

The luminometer can be used in various modes, including the solo instrument with parameter input through the keyboard, and the results read directly from the digital display. An alternative is to control the instrument with a PC, with hard copy of the digital results made available through a high speed matrix printer or continuous analogue results printed on a chart recorder. Four modes are available in measurement recording: continuous recording, peak height, rate (kinetic) measurement and integration, and can be pre-set. The computer-designed optics and highly sensitive, overload protected, PM tube provide an instrument sensitivity down to 0·4 femtomoles of ATP assay using an approved monitoring reagent. The dynamic range is six to seven decades, which means that a considerable spectrum of analyte concentrations can be processed without the need to serially dilute.

The system can be operated from one of several software programs, which enable sequences of commands to effect automation for assay protocols which can include *in situ* reagent addition and sample mixing and selection of the print-out format. An enzyme assay program is an optional package which can be used to carry out a variety of enzyme assays and includes a special program for creatine kinase assays with sample and patient identification. Phago-programs used with suitable PCs allow up to 25 phagocytosis samples to be continuously and sequentially measured, and other special programs, such as the terminal emulator and LIA curve fitting programs, are available. Assay programs can be stored either in an optional EEPROM memory or on a diskette.

*Further information from Eric Smith, Bio-Orbit (UK) Ltd, Crown House, King's Road West, Newbury, Berkshire RG14 5BY, UK. Tel.: 0635 46936; fax: 0635 37335.*

### 'Weighing the Right Way with Mettler'

A 20 page booklet from Mettler indicates the most important points that must be noted when working with micro, semimicro and analytical balances if weighing results of high quality are required. After some brief pointers concerning the location and the proper operation of the balances, the disturbing influences due to the surroundings on the weight determination are discussed in detail. Many of the influences are recognizable by a slow change in the weight display (drift).

Since correct interpretation of the technical data is also of immense importance in the assessment of a weighing result, the most common technical terms are explained at the end.

*The brochure is available from Mettler Instruments AG, CH 8606 Griefensee, Switzerland.*

### Programmable, precision dispensing robot

Iwashita's new Autoshooter-7 ASC 7000 is now available in the UK from Hakuto International. Designed for use in industrial processes, the highly sophisticated system is well suited to many dispensing applications. Capable of performing smooth, fast metering of a wide range of liquids in linear or circular modes, the ASC 7000 can be easily progammed for fully automated operation.

The standard system comprises of a robot controller and X-Y stage, to which a wide variety of dispenser heads can be fitted. Modular construction allows customization to many different applications. For example a compact table-top configuration is available to create a stand-alone system, or the controller and X-Y stage can be separated for incorporation into other equipment or production lines.

The robot controller is 1300 step programmable, with a facility to save the input data to disk. Smooth dispensing operations in linear, circular, copy and surface coating can be performed via PTP and CP control methods.

A number of different dispense controllers are also available. This enables selection of shot time ranges from 0·001 to 99·99 s at shot volumes as low as 0·0001 ml. All dispense controllers incorporate a vacuum transducer and are capable of handling a wide range of liquid viscosities. Additionally, the programmable controller can store up to eight different shot times, input by the 10 key pad or by teaching.

*Details from Hakuto International (UK) Ltd, Eleanor House, 33–35 Eleanor Cross Road, Waltham Cross, Hertfordshire EN8 7LF, UK.*

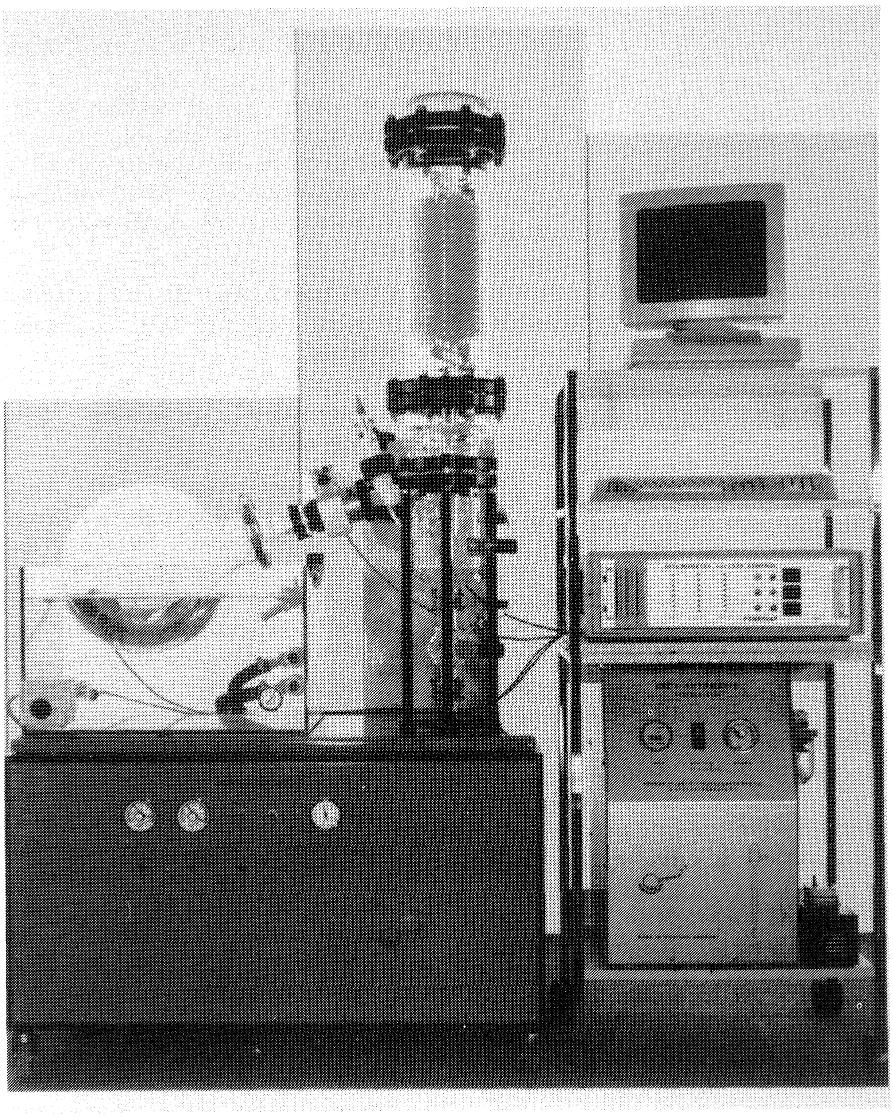

*The TOM POWERVAP high-performance robotic rotary evaporators refill and empty their flasks automatically, running 24 hours a day and maintaining a vacuum-accuracy to 0·1 mbar. The rotating seals of the POWERVAP and TOM-VAP-2 evaporators will perform for an unparallelled duration of 20 000 hours. Details from Safelab Systems, 62 Prince Street, Bristol BS1 4QD, UK. Tel.: 0272 393413.*

### Automated evaporator

The TurboVap Automated Evaporation Workstation brochure describes a faster alternative to conventional evaporation methods and provides an explanation and diagram for the patented technology behind the TurboVap. It has graphs and tables that show adjustable evaporation rates and comparative recoveries and reproducibility of pesticides and semi-volatile matrix spikes. The TurboVap Workstation automatically and independently controls the evaporation of each of up to six samples, performs an optional solvent exchange and stops the evaporation process at a pre-set endpoint. The TurboVap is compact and self-contained and can be safely operated on a laboratory bench. The TurboVap is available in two sizes—the 50 ml model will accommodate up to six samples (<50 ml) simultaneously, while the other model accommodates up to six large samples.

*Details from Zymark Corporation, Zymark Center, Hopkinton, Massachusetts 01748, USA. Tel.: 508 435 9500; fax: 508 435 3439.*

### Field emission microscope

Jeol has introduced a field emission version of the JSM 6400 electron microscope for specialized materials science research. Designated the JSM 6400F, the new scanner offers 1·5 nm resolution on samples up to 8 in. across, at magnifications ×10 to ×500 000. It is ideal for semiconducting and insulating samples and particle analysis on materials such as magnetic tapes where particle details 0·1 μm across may need to be resolved. 1 kV steps in accelerating voltage cater for uncoated or non-conducting specimens. The model has an advanced high resolution framestore. Frame averaging and integration help to improve the weakest images. The real-time display can be compared with up to four memorized images. Image processing also includes histogram display, table manipulation, grey scale contraction and expansion, gamma and log amplification. Processed images can be photographed through the recording CRT or electronically stored.

The operator has both manual and full keyboard control of column parameters, accelerating voltage and alignment settings, autofocus, contrast and brightness, and astigmatism correction.

The specimen chamber, with specimen exchange airlock, has a fully eucentric goniometer stage to handle the large specimens up to 8 in. which can be rotated through 360° and at tilt angles between −5° and 90°.

*Further details are available from Jeol (UK) Ltd, Jeol House, Grove Park, Colindale, London NW9 0JN, UK.*

### Portable PPM oxygen analyser

The Teledyne Model 311C is a portable parts-per-million oxygen analyser which has a CENELEC-approval rating of EExibIICT4. Weighing just 6 lb., the Model 311C will spot-check gas phase ppm $O_2$ in a variety of quality assurance and process monitoring applications.

The Model 311C features an integral rechargeable battery, which powers the analyser for up to 30 days between charges. The Model 311C also features four measuring ranges: 0–10, 0–100, 0–1000, 0–10 000 ppm $O_2$; a special span range for air calibration; and an easy-to-read analogue meter.

The oxygen sensor is Teledyne's patented Micro-fuel cell. This maintenance-free electrochemical sensor features an absolute zero and an output that is linear with respect to oxygen concentration. No zero gases are required and atmospheric air (209 500 ppm $O_2$) can be conveniently used for calibration.

*Further information from Teledyne Analytical Instruments, The Harlequin Centre, Southall Lane, Southall, Middlesex UB2 5NH, UK.*

### The LC analyst expert methods development system

Perkin-Elmer has developed an integrated, computer-controlled LC system designed specifically for computer-aided chromatographic research and methods development. The LC Analyst Expert Methods Development System includes the Series 620 quaternary solvent system, the LC-235 high sensitivity, diode array detector, the ISS-200 autosampler and LC Analyst software for instrument control and integration. Methods Development Software (IMDS) packages include:

LC Starting Point to aid selection of column and initial mobile phase conditions.

Perkin-Elmer Solvent Optimization Software (PESOS), which empirically tests a region of solvent space and helps determine regions of best separation or areas of separation ruggedness. DryLab Plus helps predict the best separations via simulation. LC View Software to complement chromatographic data from DryLab or PESOS with Peak Purity and Spectral Identification for increased confidence in separation.

REFLEX, a data-based management software package for post-run manipulation, interpretation or correlation of data files.

The system offers a simple user interface with mouse control and automatic start-up and shut-down.

*For further information contact Perkin-Elmer Ltd, Maxwell Road, Beaconsfield, Buckinghamshire HP9 1QA, UK. Tel.: 0494 676161.*

### Polarimeter cells

Optical Activity now offer the following polarimeter cells:

*All-glass cell*

All-glass Polarimeter cells are again becoming popular. Optical Activity's new G7 sample cells come in either 100 mm or 200 mm pathlength with dual filling tubes to make them safe and simple to fill. Samples are poured directly into the cell and come into contact only with glass, so they are suitable for use with acids and corrosive solutions.

*High-precision PTFE cell*

The PTFE cell is ideal for use with acids, and where sample volume is limited. The PTFE cell is available in pathlengths of 25 mm and 50 mm. Sample volumes are only 0·5 ml and 1 ml.

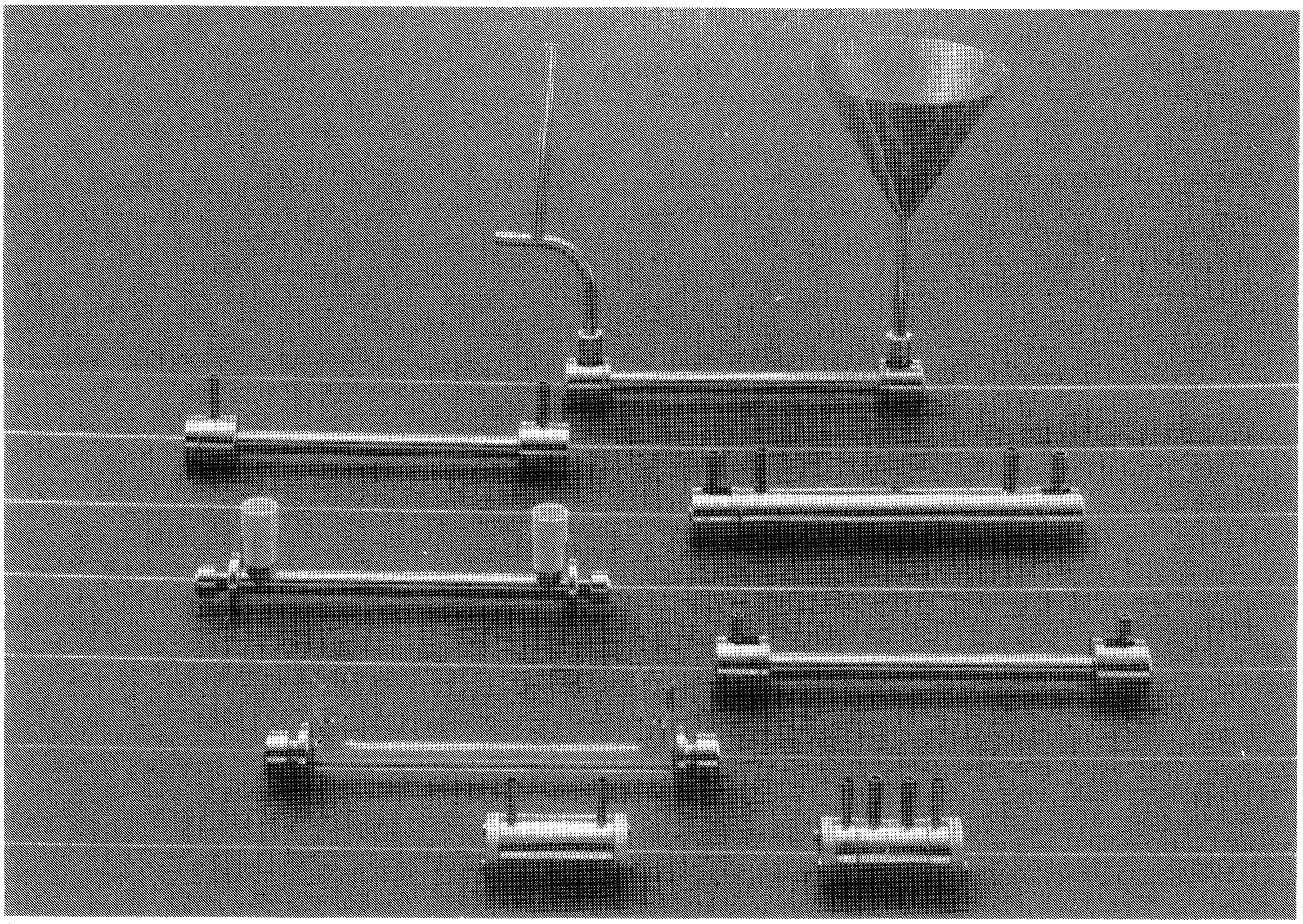

*The five polarimeter cells recently added to Optical Activity's range.*

*Small-volume, temperature-controlled cell*

These cells allow constant temperature continuous measurement of dense samples, such as unrefined sugar solutions. Standard cell pathlengths are 25 mm and 50 mm, but pathlengths of 5 mm and 10 mm can also be supplied. Optical Activity have developed a unique thermostatable shoe which ensures high measurement accuracy, even with short pathlength cells.

*Funnel cells*

In busy laboratories with high sample throughput or in process situations where results are required quickly the speed of sampling can be critical. A stainless-steel 'funnel' cell has been developed to meet this need. It is supplied complete with an antisyphon tube and only requires connecting to a suitable waste pipe. The cell is ideal for use where sample volume is not a problem, and allows up to 180 samples to be measured in an hour.

*Cell-selection chart*

With such a wide choice of cell types, sizes and materials, Optical Activity have produced a cell-selection wallchart which is available free. The chart gives full details of all Optical Activity cells with technical information and selection advice.

*Further information from Optical Activity Ltd, Bury Road Industrial Estate, Ramsey, Cambridgeshire PE17 1NA, UK. Tel.: 0487 813913.*

### Flow injection analysis

More than 2000 articles covering flow injection analysis have been published.

Tecator has recently published the *FIAstar Flow Injection Analysis Bibliography Supplement 1987/88*. The bibliography contains 500 references from scientific journals and is organized in the same way as the original *1974–1984 Bibliography* and the *1985* and *1986 Supplements*, with a cross-index covering 255 species determined with FIA, tables for different application areas and instrumental techniques used and a keyword index. All authors are listed alphabetically. Almost 90% of the references are application oriented with environmental analysis/water testing as the single most important application area.

*For further information contact Tecator AB, Box 70, S 263 21 Höganäs, Sweden. Tel.: 46 42 42330; fax: 46 42 40349.*

### Jet mixing head

The capabilities of the IKA Ultra-Turrax dispersing and emulsifying equipment have been extended with the introduction of a jet mixing head. The W80SMK jet mixing head creates an intensive vortex since the top-to-bottom flow of material is concentrated within a small 80 mm circumference. This enables normal mixing and dissolving times to be reduced, as well as eliminating aeration of the product.

The W80SMK jet mixing head is for use with either the IKA Ultra-Turrax T50 or T50 DPX unit. The T50 high-speed dispersing/emulsifying unit is designed for use with free-flowing product. It features an infinitely variable, electronically controlled drive which ensure constant motor speeds. The T50 unit is a general laboratory workhorse. Where there is a risk of fire or explosion, Sartorius is able to supply an intrinsically safe model: the T50 DPX unit.

Dependent on drive speed and product, the W80SMK can achieve a circumferential speed up to 10 000 rev/min (20·9 m/s) and has an operating range of 1 to 30 l at an immersion depth of 350 mm. The head is ideal for batching in gas or liquids, for lump-free suspending of difficult to dissolve powder or for loosening sedimented and already hardened substances.

*For further information contact Peter Butler, Sartorius Ltd, Scientific Division, Longmead Business Centre, Blenheim Road, Epsom, Surrey KT19 9QN, UK. Tel.: 03727 45811; fax: 03727 20799.*

### Cholinesterase assay

Accurate, precise and easy determination of cholinesterase activity can be obtained by means of the pH-stat method, which directly measures the hydrolysis of choline esters caused by the enzyme at a constant pH value. The pH-stat method takes only 6 min to perform, during which the reaction can be followed on a CRT screen. Result facilities include calculation of specific activity, curve of added volume versus time, curve of pH value versus time, statistics, etc.

Compared to spectrophotometric methods, pH-stat does not require buffers or substrates foreign to the enzyme, and it is not subject to errors from colour interference. Different types of cholinesterase can be determined specifically by using appropriate substrates.

The TitraLab 11 High Performance Titration Laboratory from Radiometer Analytical A/S is designed for pH-stat work, and it offers automatic calculation of results. The system measures the pH value of the sample 40 times per minute with a resolution of 0·001 pH and adjusts the continuous addition of alkali acordingly. The volume of added alkali is determined with a resolution of 0·1 µl. This gives excellent maintenance of a constant pH value and a reliable real-time kinetics curve for rate calculations.

*For further information contact Radiometer Analytical A/S, Krogshojvej 49, DK 2880 Bagsvaerd, Denmark. Tel.: 45 31696311; fax: 45 44490011.*

### Optimization of HPLC gradient separations

One of the most difficult problems facing the HPLC methods development laboratory is the optimization of gradient separations. Gradient range, composition, steepness and duration are parameters added to the features of isocratic HPLC, i.e. column performance, column and mobile phase selectivity and flow rate optimization.

Until now, the calculations involved in predicting gradient resolution from experimental data have been dauntingly complex. The end result is that most gradient HPLC separations are developed after time-consuming repeated experimental runs.

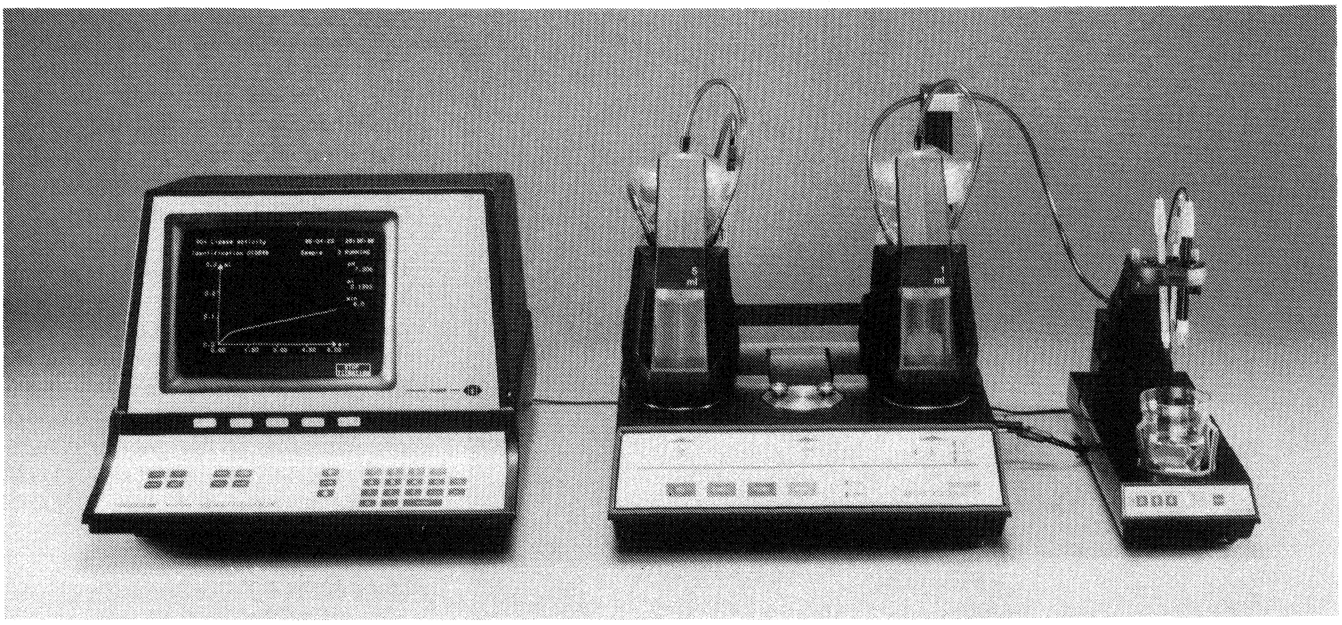

*Radiometer Analytical A/S's cholinesterase assay, which is documented in a 3 page application note describing the apparatus settings, reagents, sample preparation and typical results with statistics. The note 'Cholinesterase activity, computerized method' is available free from Radiometer.*

DryLab G allows the user to develop a final gradient elution procedure in the space of a few hours. This software system takes the user through methods development following the same approach used in DryLab I: first optimize retention and then fine-tune column parameters. DryLab G makes it easy to see exactly what happens as gradient conditions are changed.

*Further details from Autoscribe Ltd, 7 Hawkes Close, Wokingham, Berkshire RG11 2SZ, UK. Tel.: 0734 787917; fax: 0734 773867.*

### TOC network for waste-water treatment

Tennessee Eastman Company (TEC), in Kingsport, USA, the chemical manufacturing division of Eastman Kodak, have recently completed a US$80 million modernization of their waste-water treatment plant and storm sewer discharge system. The manufacturing site produces intermediate and fine organic chemicals, fibres and plastics.

The new facility incorporates 11 Ionics 6800 process TOC monitors to analyse storm sewer discharge for organic materials, including solvents and the products themselves. By measuring the organic carbon in this way, plant chemists can predict the effect of the waste-water on the treatment plant and forestall any difficulties.

The 11 monitors are networked via a mainframe computer covering eight sites on the complex. In addition, two further Ionics 6800s monitor the carbon waste load and effluent quality to and from the waste-water treatment plant. From a central control room, an analyst can check storm sewer discharge at 19 points within these sites over a total area of 2 square miles.

If a stream result indicates a high alarm, the Ionics 6800 can be 'locked' on the stream from the control room to monitor the magnitude of the incident and to divert the stream to a holding tank to provide time for corrective plant action.

The computer logs the data from each site and performs relevant statistical record keeping. As a back-up, each analyser includes a recorder to log locally each output, to provide the data independently of the computer.

Model 6800s include a failsafe alarm package to prevent erroneous data in the event of a loss of instrument air pressure, carrier gas pressure, or sample flow. Similarly, should the acid supply fail or furnace temperature change, then the operator is notified.

*Further details are available from Ionics UK Ltd, Carrington Business Park, Carrington, Urmston, Manchester M31 4DD, UK.*

### Automated evaporator

The TurboVap LV is an automated low volume (1–30 ml) evaporator. It uses nitrogen to create a patented vortex action in the sample. It is capable of evaporating 10 ml of $MeCl_2$ in less than 15 min at 38 °C, or 10 ml of Hexane in less than 9 min at 52 °C. The TurboVap LV can simultaneously evaporate from 1 to 50 samples to dryness and regulates the bath temperature from ambient to 90 °C. The workstation is compact and self-contained and operates on the laboratory bench instead of in a hood. The TurboVap LV has a unique manifold design that conserves gas during operation and does not use expensive glassware. It uses standard 16 × 100 or 20 × 150 mm tubes. The TurboVap LV can also be interfaced to the BenchMate Workstation, which allows automated sample preparation to be combined with automated evaporation for pharmaceutical sample preparation, forensic analysis, drug meta-

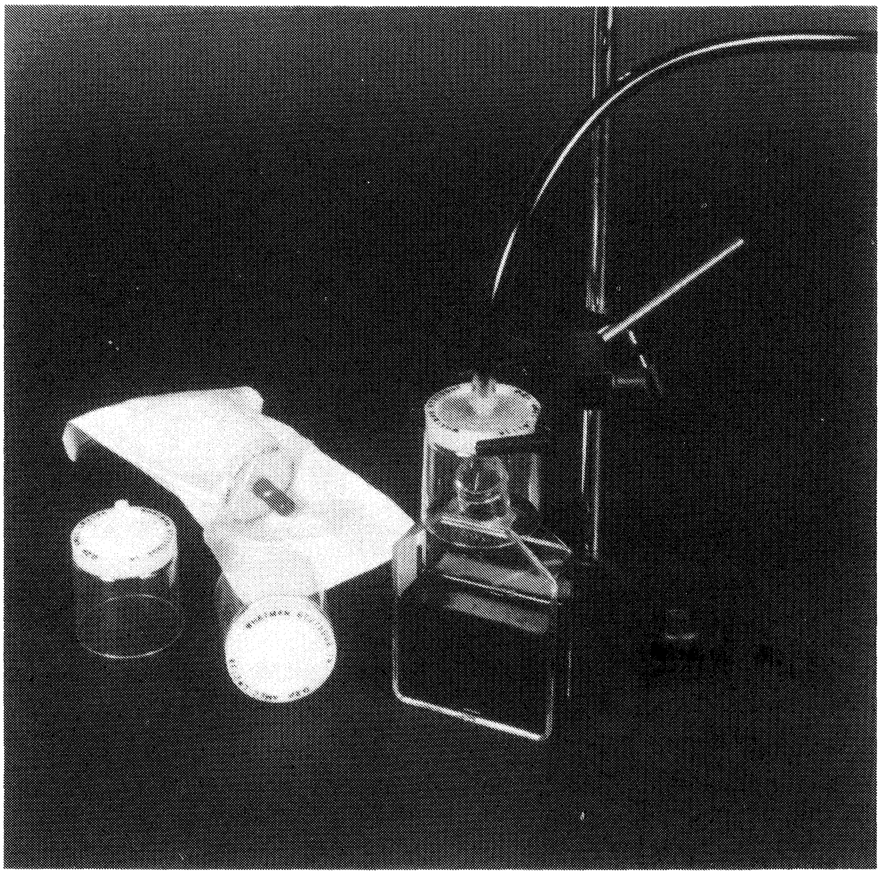

*The Polydisc AS for sterilizing and filtering such aqueous solutions as tissue culture media, antibody and enzyme solutions and laboratory water supplies. The advantage of the filling bell is that it covers the neck of the receptacle and minimizes the risk of extraneous contamination. This disposable device features a low-binding membrane made of asymmetric mixed esters of cellulose, and a glass microfibre prefilter to extend the membrane life and allow filtration of larger volumes. There is a choice of 0·2 and 0·45 μm pore sizes, which provide high flow rates and fast sample throughput. Polydisc AS microfiltration devices are presterilized by irradiation and supplied in sealed bags. The devices are available from Whatman, Springfield Mill, Maidstone, Kent ME14 2LE, UK.*

bolism studies, toxicology studies and other life science and general laboratory applications.

*Contact Sharon Correia, Zymark Corporation, Hopkinton, Massachusetts 01748, USA, for more information.*

### Batch recipe management software

Rotork Instruments (formerly Protech Instruments) have launched a batch recipe management software package that will simultaneously run numerous batches in several multi-stage plants, whilst holding additional recipes in reserve, ready for manufacture as plant becomes available.

Offered in two versions, it extends the sequencing capability already available for Toshiba Tosdic advanced distributed control systems (DCSs), enabling a 'unit operations' sequence strategy to be introduced, i.e. each unit operation can become a stage in a more extensive manufacturing process and can be held in a library to be linked in various ways in accordance with the user's requirements.

In its simple form it is designed for the small scale Tosdic 243D DCS, with a more complex version for the large-scale Tosdic 247 system.

*Details from Rotork Instruments, 241 Selbourne Road, Luton, Bedfordshire LU4 8NP, UK. Tel.: 0582 596181.*

### FP800HT thermosystem

The research and development of new materials often calls for exact knowledge of their thermal behaviour. In quality assurance or production monitoring, experimental results are compared with a standard.

The FP800HT Thermosystem from Mettler, with five measuring cells for various analytical procedures, is an efficient and appropriate tool for the numerous analyses in day-to-day operations in this field. In comparison with the earlier thermosystem, the temperature range of several cells has been extended by 50 °C to 375 °C.

The FP80HT Processor is the central unit of the FP800HT Thermosystem; it functions as the control and communication device of the system. Any one of five measuring cells can be attached to it: the FP81HT measuring cell for melting, boiling and cloud points; the FP83HT measuring cell for dropping and softening points; the FP82HT/FP84HT thermal microscopy hot stages; or the FP85 measuring cell for DSC (differential scanning calorimetry).

The operation of the system is simple and flexible. The analysis is started at a keystroke after entry of the required parameters. The FP80HT then controls the attached measuring cell according to the set method.

Calculation of the results from the experimental data is automatic. An attached printer generates a record, and an analogue recorder can be used to plot the measured signal during the analysis. An interface built in as standard also allows bidirectional data interchange with a computer or an LIMS.

*Details from Mettler-Toledo AG, CH 8606 Greifensee, Switzerland.*

### Column for physiological analyses

Beckman have developed a 10 cm high-performance column for physiological methods in the Beckman System 6300 amino-acid analyser. The lithium column replaces the Beckman 20 and 25 cm lithium columns, and provides increased col-

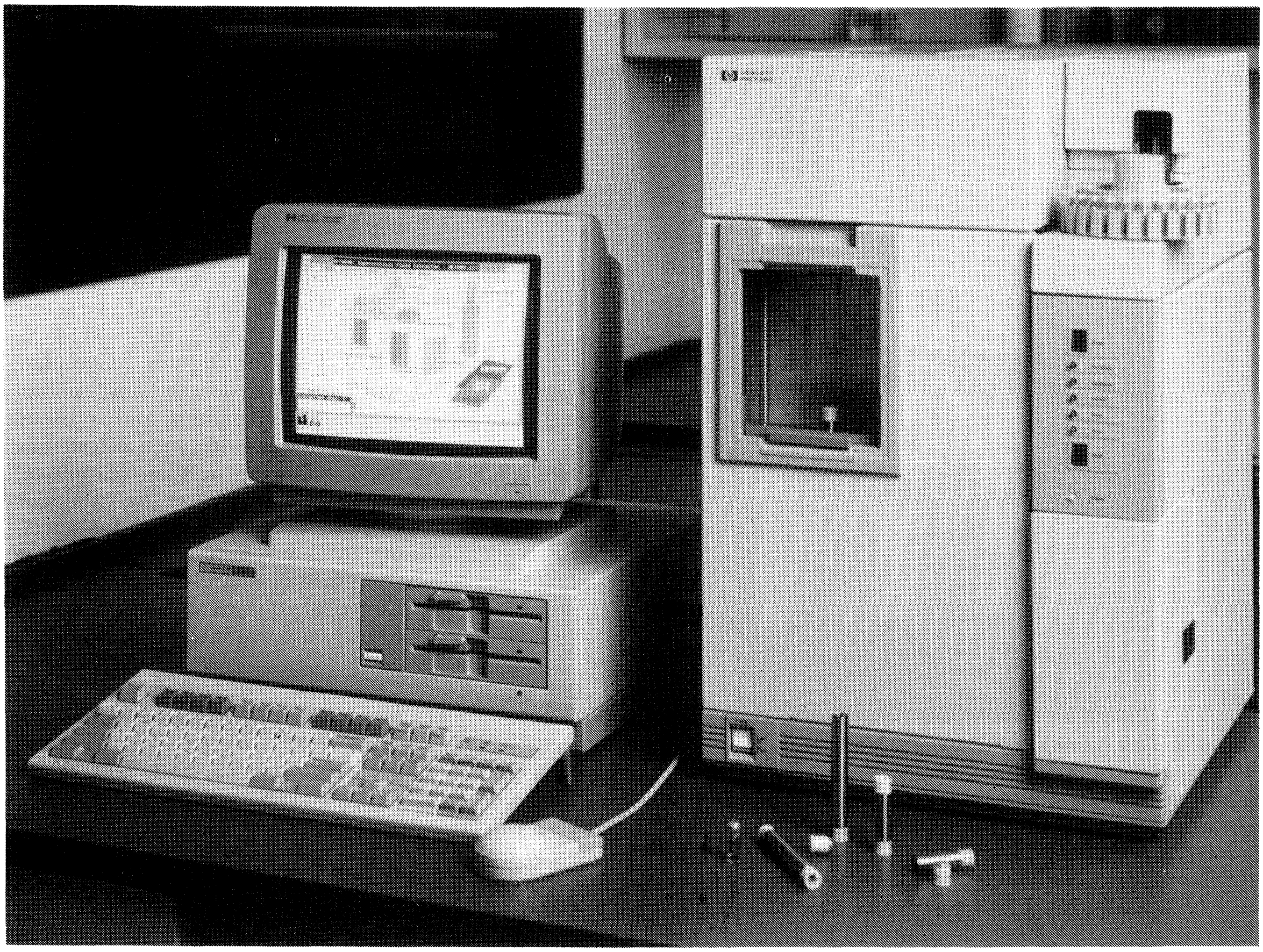

*Hewlett-Packard Company has introduced a graphics-driven, computer-controlled supercritical fluid extractor (SFE). The HP 7680A SFE is HP's first instrument dedicated to sample preparation, and is believed to be the first sample preparation instrument to use an extensive graphics interface to facilitate operation of the sample preparation procedure. Full information from Verena Haller, Hewlett-Packard S.A., 150 route du Naut-d'Avril, CH 1217 Meyrin 2, Switzerland.*

umn life, improved resolution and the convenience of using a single column for different analyses.

The total system, including the System 6300, the 10 cm column and buffers, facilitates repeatable, reliable separations of up to 52 components in 2 hours.

*Details from Beckman, Progress Road, Sands Industrial Estate, High Wycombe, Buckinghamshire, UK. Tel.: 0494 441181.*

### Gensym in Europe

Gensym Corporation has announced the establishment of the company's first European subsidiary: Gensym GmbH in Munich, FR Germany. This new subsidiary will be directly responsible for sales and support of Gensym's Real-Time Expert System, G2, in FR Germany and other countries, as well as providing support for Gensym's existing distributors and value-added resellers in Europe.

Based in Cambridge, Massachusetts, USA, Gensym revenues are expected to reach approximately $10 million in 1990, up approximately $5 million from 1989.

G2 is currently used in more than 400 applications in a range of industries including: aviation, aeronautics, and defence; computer-integrated manufacturing; chemical, petroleum, oil and gas; iron, steel, and metals; pulp and paper; energy, power and utilities; and environmental systems.

*Further information about G2 and other products from Gensym Corporation, 125 Cambridge Park Drive, Cambridge, Massachusetts 02140, USA. Tel.: 617 547 9606; fax: 617 547 1962. Or from Gensym GmbH, Leopoldstrasse 28a/II, D 8000 München 40, FR Germany.*

### Evaporators and extractors

A catalogue describing high-speed analytical evaporators, extractors and accessories is being offered by

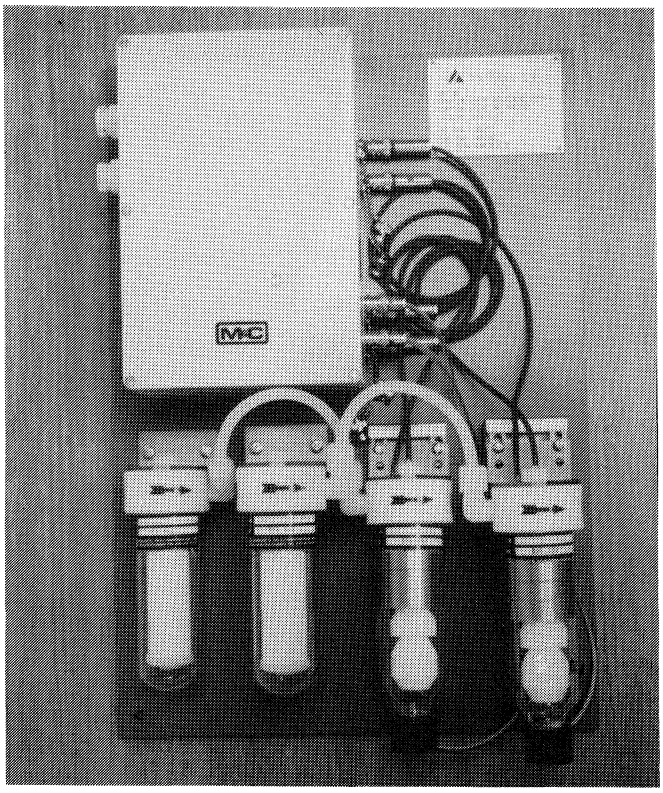

*Anatrol Ltd are offering two electrostatic filters for the removal of aerosols from gases. The filters are available in single or dual channel versions. Each channel has a pre-filter, and the electrostatic unit incorporates an automatic drain. The electronics unit has safety trips for misuse and fault indication. The main application for the filters is as an integral part of a permanently mounted sample system for flue gas analysis such as for desulphurization plants in power stations. Data sheets for the two filter units may be obtained from Anatrol Ltd, Unit 10, Hampton Heath Industrial Estate, Near Malpas, Cheshire SY14 7LU, UK.*

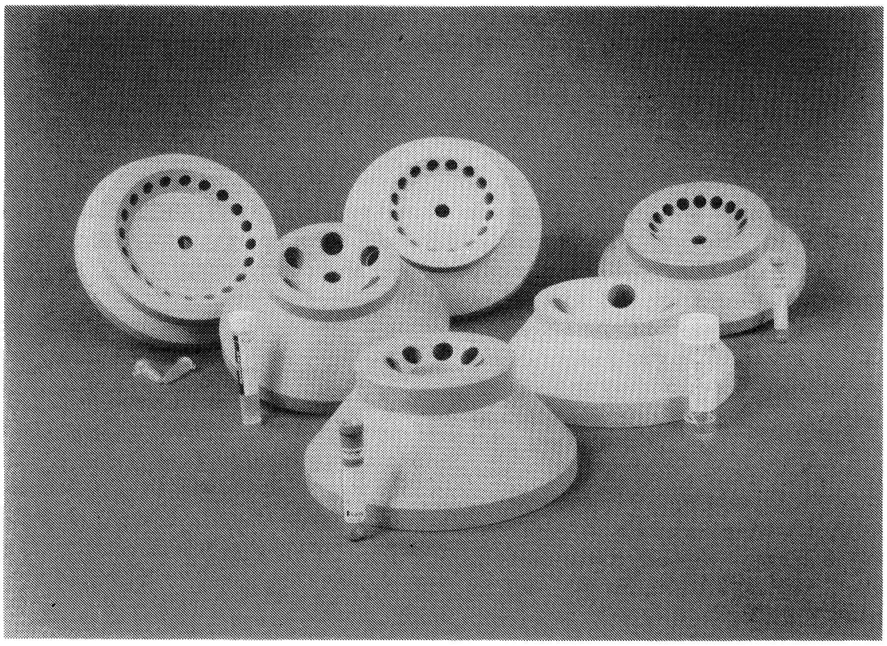

*Centrilab's custom heads.*

Organomation Associates Inc. of Berlin, Massachusetts, USA. The Organomation Catalogue 611 describes a full line of high-speed analytical equipment, including 12 to 36 position N-EVAP, and 49 to 100 position MULTIVAP evaporators; S-EVAP models for handling up to eight sets of Kurderna-Danish glassware; AQUA-VAP analytical water evaporators; and compact ROT-X-TRACT rotary analytical extractors for solids and the ROT-X-TRACT-L for liquids. Complete with product descriptions, photographs, specifications and a broad range of accessories, such as test-tube racks, clamps, needles (cannulae) and pipette adapters, the catalogue carries useful suggestions for selecting the appropriate combination of equipment and accessories. Electrical voltages and plugs for each country are available.

*Copies from Organomation Associates, Inc., Neal L. McNiven, General Manager, 266 River Road West, Berlin, Massachusetts 01503, USA. Tel.: 508 393 2602; fax: 508 838 2786.*

### Bench top head swap

Centrilab are offering a range of custom heads to suit most sizes and shapes of sample tube, for the CEP Series of Centrifuges. Using their novel design of rotor milled from polypropylene, Centrilab are now able to provide a rotor to suit most applications. Typically this would relate to VACUTAINER, MONOVETTE or Reaction Vials in various sizes from 1·5 ml to 15 ml. Custom rotors of this type ensure that CEP Centrifuges can now provide flexible, high performance as a sole resource in small laboratories.

The polypropylene rotors are easily removable and have a high degree of dimensional stability at temperatures between −20 °C to +125 °C. This allows for rapid changeover of heads and refrigeration, or sterilizing, as required.

*Details from Centrilab, Kingsbury House, Fridays Cross Mews, Christchurch Road, Ringwood, Hants BH24 1DG, UK. Tel.: 0425 480455.*

